# Antibiotic growth promoters and waxy corn enhance broiler growth performance through starch digestibility and microbiota modulation in the crop and ileum

**DOI:** 10.1016/j.psj.2025.105288

**Published:** 2025-05-10

**Authors:** Yanwei Xu, Yong Xiao, Chunxiao Ai, Yihong Huang, Huajin Zhou, Jianhui Li, Jianmin Yuan

**Affiliations:** a State Key Laboratory of Animal Nutrition and Feeding, College of Animal Science and Technology, China Agricultural University, Beijing, 100193, China; b College of Animal Science, Shanxi Agricultural University, Taigu, 030801, China

**Keywords:** Starch digestibility, Growth performance, Microbiota, Virginiamycin, Broiler

## Abstract

This study aimed to investigate the internal connections between starch digestion properties and microbial changes in the gastrointestinal tract of broiler chickens. A total of 240 male AA+ broilers (21-day-old) were allocated to 4 treatments of regular and waxy corn with and without virginiamycin (6.4 g/t) in a 2 × 2 factorial arrangement. On day 35, growth performance and apparent starch digestibility were determined, and hormones in the serum and microbial profiles in the crop and ileum were analyzed. The results indicated that dietary starch was primarily digested in the jejunum of broiler chickens, and broilers receiving waxy corn exhibited significantly greater digestibility of both total starch and amylopectin in the distal jejunum, along with an improved feed-to-gain ratio **(F/G)** and European performance index **(EPI)**, compared with the regular corn group (*P<* 0.05). Notably, virginiamycin significantly enhanced the amylose digestibility in the distal ileum and improved the F/G ratio (*P<* 0.05). Microbial analyses revealed that corn type did not affect microbial diversity in the crop and ileum. However, virginiamycin significantly reduced alpha diversity in the crop (Chao1: 129.63 vs*.* 191.14, *P* < 0.05) and ileum (observed species: 137.23 vs*.* 316.43, *P* < 0.05), and elevated *Lactobacillus* abundance (positively correlated with amylose digestibility, *P* < 0.001). In summary, waxy corn enhanced jejunal starch hydrolysis via its high amylopectin content, whereas virginiamycin promoted amylose digestion in the distal small intestine by selectively enriching the starch-degrading microbiota.

## Introduction

Starch, which constitutes approximately 50 % of the energy in poultry diets on a dry matter (**DM**) basis, serves as a critical energy source that is primarily hydrolyzed into glucose through enzymes secreted by animals (Svihus, 2014a). Digestibility plays a key role in feed efficiency and growth. Starch digestion occurs sequentially through the gastrointestinal tract, with approximately 65 % of digestion reaching the terminal duodenum, 85 % reaching the terminal jejunum, and up to 95 % reaching the terminal ileum (Riesenfeld et al., 1980). In addition to host-secreted digestive enzymes, the gastrointestinal microbiota contributes to starch digestion (Wang et al., 2024). Despite the lack of teeth, poultry possess a unique digestive structure (crop). The crop functions as the primary site for the initiation of digestion in the avian alimentary tract, where the softening of feed and fermentation processes begin (De Cesare et al., 2020; Kristoffersen et al., 2022). The predominant microorganism in the crop, *Lactobacillus*, can perform the preliminary fermentation of dietary nutrients and produce organic acids (Saxena et al., 2016). However, limited research has been conducted on how crop pretreatment with dietary starch affects digestibility in the small intestine. In addition to microbial activity in the crop, the microbiota within the intestines can influence, to some extent, the digestion of dietary starch in the host.

The structure of starch, particularly the amylose-to-amylopectin ratio, significantly influences *in vivo* and *in vitro* digestibility (Van Hung et al., 2016; Guo et al., 2017), with amylopectin being digested more rapidly than amylose (Ao et al., 2007). This difference arises from hydrogen bonds between the glucose chains in amylose, which makes the molecule more resistant to enzymatic breakdown (Lv et al., 2021). In contrast, the shorter linear chains of amylopectin make it more accessible to amylase (Syahariza et al., 2013). Waxy corn starch typically consists mainly of amylopectin (0 to 2 % amylose), whereas regular corn starch contains approximately 15 to 30 % amylose (Van Hung et al., 2016). Amylopectin is rapidly degraded by α-amylase in the gastrointestinal tract of monogastric animals, leading to higher blood glucose and insulin levels (Yang et al., 2016) and improved growth performance (Ma et al., 2019).

Conversely, amylose is generally considered a slow-digesting starch, which is hydrolyzed in the hindgut and fermented by microorganisms (Yang et al., 2022). Although amylopectin is digested more rapidly, its digestion may remain incomplete in the upper gastrointestinal tract due to the reliance on pancreatic amylase, which reaches the duodenum via reflux (Svihus, 2014b), and the faster intestinal emptying rate in broilers. Therefore, we hypothesized that distinct differences would emerge in the hydrolytic and fermentation properties of amylopectin and amylose in the post-intestinal tract, which could influence the subsequent energy utilization in broilers.

Beyond intrinsic starch characteristics, exogenous modulators, such as antibiotics, can significantly affect nutrient utilization. Antibiotics, such as virginiamycin, which are traditionally used as growth promoters in feed, regulate gastrointestinal microbial communities and potentially affect growth performance (Payen et al., 2023; Fonseca et al., 2024). However, it remains unclear whether antibiotics induce changes in microbiota composition, particularly in the crop (initial site) and ileum (final site), and how such changes might affect starch digestibility. Therefore, we regulated the type of corn starch in the diets and altered the microbial communities in the crop and ileum of broilers using antibiotics, to investigate the energetic benefits of poultry on microbial changes across different digestive tract sites and starch digestion. These findings provide critical insights into the formulation of precise nutritional strategies for poultry production.

## Materials and methods

### Ethics statement

The trial was conducted at the Zhuozhou Poultry Nutrition Research Base of the China Agricultural University (Hebei, China). All animal procedures adhered to the Beijing Regulations of Laboratory Animals (Beijing, China) and were approved by the Laboratory Animal Ethical Committee of China Agricultural University (approval number: Aw42205202-1-02).

### Birds and bird housing

Male AA+ broiler chicks (1-day-old) were obtained from a commercial hatchery (Beijing Poultry Breeding Company, China) and randomly allocated to 24 battery cages on the same floor, each furnished with 3 nipple drinkers. The birds were raised under controlled light conditions (23L:1D, 30 lux during the first 2 days followed by 16L:8D, 10 lux until the end of the experiment) and temperature (starting at 33 °C and reduced daily to reach 21 °C by day 25). Feed and water were provided ad libitum. All birds were fed a commercial diet for 1 to 20 days.

### Experimental treatments

At 21 d of age, 240 broilers with uniform body weight were randomly assigned to a 2 × 2 factorial arrangement comprising corn type (regular or waxy) and virginiamycin addition (or not). This resulted in four dietary treatments (Table 2), each containing six replicate pens of 10 birds. Waxy corn and regular corn were purchased from Bonong Poultry Industry Co., Ltd. (Hebei, China) and ground through a hammer mill equipped with a 2.5-mm screen. The nutritional composition of waxy corn and regular corn was presented in Table 1. Diets were formulated to meet or exceed the nutritional requirements of the broiler chickens (NRC, 1994), and starch composition and content in the diet were detected (Table 2). The experimental diets were fed from 21 to 35 d. A digestibility assay was conducted from 32 to 35 d of the experimental period using titanium dioxide (**TiO_2_**) as a digestibility marker. Due to the delicate characteristics of the ingredients, diets were pelleted with a conditioning temperature not exceeding 75 °C.

**Table 1:** Chemical composition of waxy corn and regular corn (air-dried basis, %).

Item	Waxy corn^1^	Regular corn
Dry matter	89.49	88.98
Crude protein	8.64	8.25
Crude fiber	2.00	1.83
Ether extract	3.90	3.45
Ash	1.30	1.38

1 Waxy corn variety:Xianda Nuo 001.

**Table 2:** Ingredients and nutrient composition of the diets (air-dried basis).

Ingredients (%)	Corn-N	Corn-ABV	Waxy-N	Waxy-ABV
Regular corn	57.10	57.10	-	-
Waxy corn	-	-	57.10	57.10
Soybean meal, CP 46.80 %	30.00	30.00	30.00	30.00
Corn gluten meal, CP 63.50 %	3.00	3.00	3.00	3.00
Wheat flour	2.00	2.00	2.00	2.00
Soybean oil	4.00	4.00	4.00	4.00
Calcium hydrogen phosphate	1.40	1.40	1.40	1.40
Limestone	1.20	1.20	1.20	1.20
NaCl	0.34	0.34	0.34	0.34
Vitamin premix^1^	0.30	0.30	0.30	0.30
Mineral premix^2^	0.20	0.20	0.20	0.20
Choline chloride, 50 %	0.20	0.20	0.20	0.20
L-Lysine sulphate, 78 %	0.30	0.30	0.30	0.30
DL-Methionine, 98 %	0.20	0.20	0.20	0.20
Phytase, 1000	0.01	0.01	0.01	0.01
Ethoxyquinoline, 33 %	0.02	0.02	0.02	0.02
Virginiamycin, 8 %	-	0.008	-	0.008
Nutrients level				
Total starch^3^, %	42.73	42.31	42.68	42.65
Amylopectin, %	31.12	31.06	38.72	38.65
Amylose, %	11.61	11.25	3.96	4.00
Amylopectin/Amylose	2.68	2.76	9.78	9.66
Metabolizable energy, kcal/kg	3110	3110	3110	3110
Crude protein, %	21.34	21.34	21.57	21.57
Lysine, %	1.21	1.21	1.21	1.21
Methionine, %	0.52	0.52	0.52	0.52
Threonine, %	0.78	0.78	0.78	0.78
Calcium, %	0.86	0.86	0.86	0.86
Nonphytate, %	0.34	0.34	0.34	0.34

Corn-N, a diet based on regular corn without virginiamycin; Corn-AVB, a diet based on regular corn and inclusion of 6.4 g/t virginiamycin; Waxy-N, a diet based on waxy corn without virginiamycin; Waxy-ABV, a diet based on waxy corn and inclusion of 6.4 g/t virginiamycin.

1 The vitamin premix provided the following per kilogram of complete feed: Vitamin A, 12500 IU; Vitamin D3, 2500 IU; Vitamin E, 30 IU; Vitamin K3, 2.65 mg; Vitamin B1, 2 mg; Vitamin B2, 6 mg; Vitamin B12, 0.025 mg; Biotin, 0.0325 mg; Folic acid, 1.25 mg; Niacin, 50 mg; Pantothenic acid, 12 mg.

2 The mineral premix provided the following per kilogram of complete feed: Iron, 80 mg; Copper, 8 mg; Manganese, 100 mg; Zinc, 75 mg; Iodine, 0.35 mg; Selenium, 0.15 mg.

3 Starch content was experimentally determined.

### Growth performance measurements

Body weight (**BW**) and feed intake (**FI**) were measured on day 21 and 35 to calculate the body weight gain (**BWG**), feed-to-gain ratio(**F/G**), and European performance index (**EPI**).

### Sample collection

On day 34, blood samples were collected via wing vein puncture from fasted broilers after a 10-hour fast, and feeding was then resumed. On day 35, six birds per cage with similar body weights were randomly selected for sample collection and euthanized by an intravenous injection (1 mL per 2 kg live weight) of sodium pentobarbitone. To minimize inter-group temporal confounding, sample collection was simultaneously initiated across all four treatment groups by four teams of trained researchers. Crop contents were mixed and approximately 1.5 g was placed in a tube and stored at −80 °C; the remaining digesta were collected and freeze-dried. Intestinal contents from the distal jejunum and ileum were collected by gently finger-stripping these gastrointestinal tract segments and subsequently frozen at −20 °C for future starch and TiO_2_ content analysis. Another 2 g of the terminal ileum digesta was collected and stored at −80 °C for microbial quantification; the mucosa of each intestinal segment was scraped using a sterilized slide and immediately placed in liquid nitrogen for RNA extraction.

### Chemical analyses

Diets, crop, jejunal, and ileal digesta were dried and ground (0.5 mm sieve) before chemical analyses. DM was determined by drying the diet at 105 °C for 4 h, as per the AOAC method (AOAC, 2012).

Total starch in the diets, crop, jejunal, and ileal digesta was measured using a Megazyme assay kit (K-TSTA; Megazyme International Ireland Ltd., Wicklow, Ireland). Amylopectin and amylose in the diets, crop, jejunal, and ileal digesta were determined using a Megazyme-amylose/amylopectin assay kit (K-AMYL; Megazyme International Ireland Ltd., Wicklow, Ireland).

TiO_2_ content was quantified as described by Bautil et al. (Bautil et al., 2021). Briefly, 0.1 g of dried digesta or 0.15 g of feed was digested in concentrated sulfuric acid using a copper reaction catalyst. A 30 % w/v hydrogen peroxide solution was added during this process for TiO_2_ precipitation. Subsequently, the precipitates were removed by filtration, and the extinction values were measured at 410 nm against deionized water as a control.

### Analysis in serum

Serum samples were analyzed for glucose, triglycerides, insulin-like growth factor 1 (**IGF-1**), triiodothyronine, tetraiodothyronine, insulin, and glucagon. Glucose and triglyceride concentrations were determined by enzymatic analysis using a clinical chemistry analyzer (Bio Majesty TM, JCA-BM9130, JEOL Ltd., Akishima, Tokyo, Japan). The concentrations of triiodothyronine, tetraiodothyronine, insulin, and glucagon were measured using a radioimmunoassay device (Nuclear Instruments Factory, China). The serum IGF-1 concentration was quantified using a solid-phase, 125I-labeled immunoradiometric assay with a commercially available kit (FUJIREBIO Inc., Tokyo, Japan).

### Activity of Na⁺/K⁺-ATPase

The Na⁺/*K⁺*-ATPase activity in broiler ileal tissues was measured using a commercially available Na^+^/*K*^+^-ATPase activity assay kit (catalog numbers A070-2-2; Nanjing Jiancheng Bioengineering Institute, Nanjing, China), according to the manufacturer’s instructions.

### RNA extraction and real-time PCR analysis

Total RNA was extracted from approximately 0.05 g of mucosal samples from each broiler using TRIzol (TAKARA, Japan) and then reverse transcribed into cDNA using the PrimeScript TM RT reagent kit (TAKARA, Japan). The 10 μL real-time PCR reaction mixture contained a 1.0 µL cDNA template, 5 μL SYBR Green mix, 0.4 μL each of the forward and reverse primers, 0.2 µL ROX correction fluid (TAKARA, Japan) and 3 µL double distilled water. The PCR reaction (ABI 7500, USA) for each gene was carried out as follows: an initial step at 95°C for 30 s, followed by 40 cycles of denaturation at 95°C for 5 s, annealing at 60°C for 34 s, and extension at 72°C for 15 s. β-actin was selected as an internal reference and the relative expression of each target gene was calculated using the 2^−ΔΔCt^ method.

### Sequence analysis of microorganisms in the crop and ileum

Total microbial genomic DNA was extracted from the ileal digesta using the QIAamp Fast DNA Stool Mini Kit (Qiagen Hilden., Germany). The V3-V4 region of the 16S rRNA gene was amplified with the universal primers 341F (5′-ACTCCTACGGGAGGCAGCAG-3′) and 806R (5′-GGACTACHVGGGTWTCTAAT-3′). Read quality was assessed using FastQC software (version 0.11.5), and low-quality reads (lower than 30) and adapters were eliminated. All subsequent steps were performed using QIIME 2 (version 2019.1). Reads were processed using the DADA2 methodology to denoise sequences, correct sequencing errors, remove chimeras, and identify amplicon sequence variants (**ASV**). Default parameters were used and the read length was truncated to 260 bases. ASV with frequencies below 0.1 % were excluded. Taxonomic assignment of ASV was performed using the SILVA database (version 132) with a 97 % similarity threshold. All raw sequence datasets obtained were uploaded to the NCBI Sequence Read Archive (**SRA**) under the accession number PRJNA1227643.

### Calculation and statistical analysis

The following equation was used to calculate the apparent digestibility of total starch, amylopectin, and amylose:

$$\text{Apparent}\;\text{digestibility},\;\%=100-\left(\frac{\text{Ti}{O_{2\;}}_{\text{diet}}}{\;\text{Ti}O_{2}{\;}_{\text{digesta}}}\;\times \frac{\text{Nutrien}t_{\;\text{digesta}}\;}{\text{Nutrien}t_{\;\text{diet}}}\times \;100\right)$$

Statistical analyses were performed using SPSS software (SPSS v. 20.0; SPSS Inc., Chicago, IL). The normality of residuals and variance homogeneity were assessed for all datasets before performing statistical analyses. Data with unequal variances were transformed using an arcsine function. Two ways ANOVA was conducted to assess the main and interaction effects of diet and antibiotics. The standard deviation of the mean was also calculated to estimate the statistical variation. Bacterial diversity metrics were calculated using normalized ASV tables. Bar plots at the phylum and genus levels were generated to visualize the taxonomic distribution. Microbiota composition analyses relative to the abundance at the phylum and genus levels were performed using t-tests. Non-metric multidimensional scaling (**NMDS**) was applied to compare the microbial communities in the digesta of the crop and ileum using weighted UniFrac distances. Differences were considered statistically significant at *P* < 0.05.

## Results

### Growth performance

The corn type and supplemental antibiotics significantly affected the growth performance of broiler chickens (Table 3). Waxy corn significantly increased EPI and decreased F/G of broilers during days 21 to 35 (*P* < 0.05 or 0.01). Supplementation with antibiotics decreased the F/G ratio (*P* < 0.01) in broilers on days 21 to 35. However, no significant interaction was observed between the diet and antibiotics with respect to growth performance during the experimental period.

**Table 3:** Effect of corn types and ABV on the growth performance of broilers from 21 to 35 days.

	ABV	FI (g)	BW (g)	BWG (g)	F/G	EPI
Regular corn	-	1668±40	1915±51	1285±29	1.30±0.02	421±9
	+	1659±87	1916±58	1297±66	1.28±0.02	428±15
Waxy corn	-	1718±77	1963±67	1353±60	1.27±0.01	441±15
	+	1709±79	1958±50	1321±66	1.25±0.01	446±13
Effect						
Diet	regular corn	1663±64	1915±54	1291±49	1.29±0.02	425±12
	waxy corn	1714±75	1961±57	1337±62	1.26±0.01	444±13
ABV	-	1693±64	1937±53	1319±57	1.29±0.02	431±15
	+	1683±83	1939±60	1309±64	1.27±0.02	437±16
*P*-value						
Diet		0.108	0.075	0.068	0.001	0.003
ABV		0.774	0.910	0.685	0.017	0.333
Deit × ABV		1.000	0.895	0.361	0.835	0.871

FI, feed Intake; BW, body weight; BWG, body weight gain; F/G, feed intake / body weight again; EPI, European performance index; “+”, Inclusion of 6.4 g/t virginiamycin in the Diet (ABV); “-”, Diet without virginiamycin; *P*-value: indicates ANOVA *P*-value; *n* = 6 replicate cages per treatment.

### Starch digestibility, gene expression of glucose transporter, and serum characteristics

We attempted to measure the starch digestibility in the crop and found that digestion had just begun. Therefore, the results are only presented as starch content in the table (Table 4). The total starch content in the crop was lower than that in the feed (except for the waxy corn-without ABV group), and neither corn type nor antibiotic virginiamycin (**ABV**) had a significant effect on the total starch content in the crop. However, a significant Diet × ABV interaction was observed for the total starch content, amylopectin content, and amylopectin/amylose ratio. Specifically, in the waxy corn-without ABV group, the content of amylopectin and the amylopectin/amylose ratio in the crop were significantly higher than those in the other treatment groups (*P* < 0.05), suggesting that the degradation of amylopectin was slower in chickens fed waxy corn without ABV, particularly in comparison with those fed waxy corn with ABV. Notably, corn type also significantly affected amylopectin content in the crop and the amylopectin/amylose ratio in broiler chickens (*P* < 0.05).

**Table 4:** Content of total starch, amylopectin, and amylose in the crop chyme (g/100 g DM).

Diet	ABV	Total starch	Amylopectin	Amylose	Amylopectin/Amylose
Regular corn	-	43.52±2.02^b^	27.87±1.57^b^	15.65±2.20	1.82±0.32^b^
	+	47.10±1.63^ab^	32.26±2.27^b^	14.84±2.40	2.25±0.57^b^
Waxy corn	-	50.31±6.29^a^	39.61±6.33^a^	10.70±3.58	4.07±1.52^a^
	+	42.92±1.93^b^	30.35±3.51^b^	12.58±1.87	2.50±0.68^b^
Effect					
Diet	regular corn	45.31±2.56	30.07±2.96	15.25±2.24	2.03±0.50
	waxy corn	46.62±5.88	34.98±6.87	11.64±2.89	3.28±1.39
ABV	-	46.91±5.69	33.74±7.55	13.18±3.83	2.94±1.58
	+	45.01±2.77	31.30±2.99	13.71±2.37	2.37±0.61
*P*-value					
Diet		0.376	0.006	0.030	0.003
ABV		0.202	0.139	0.621	0.136
Deit × ABV		0.001	0.001	0.219	0.013

Within a column, means not sharing a common superscript letter are significantly different at *P* < 0.05; “+”, inclusion of 6.4 g/t virginiamycin in the diet (ABV); “-”, diet without virginiamycin; *P*-value, indicates ANOVA *P*-value; *n* = 6 replicate cages per treatment.

The types of corn and antibiotics had different effects on starch digestibility in the jejunum and ileum of broilers. In the terminal jejunum, broilers fed the waxy corn diet showed significantly higher digestibility of total starch and amylopectin than those fed the regular corn diet (*P* < 0.05). Conversely, the addition of antibiotics did not substantially affect starch digestibility in the jejunum. In the distal ileum, corn type did not significantly affect starch digestibility, although the inclusion of antibiotics in the diet significantly increased amylose digestibility (*P* < 0.05). Notably, no significant Diet × ABV interaction was observed for starch digestibility in the small intestine (Table 5).

**Table 5:** Apparent total starch, amylopectin, and amylose digestibility in terminal jejunal and ileal for broiler chickens receiving different dietary corn starches and antibiotics.

Diet	ABV	Digestibility of starch in terminal jejunum (%)			Digestibility of starch in terminal ileum (%)		
		Total starch	Amylopectin	Amylose	Total starch	Amylopectin	Amylose
Regular corn	-	92.41±3.52	91.28±5.92	93.66±3.02	98.37±0.66	98.60±0.76	96.75±0.91
	+	90.91±5.21	89.73±4.23	94.15±3.29	98.54±0.24	98.46±0.41	98.56±0.56
Waxy corn	-	95.15±2.75	96.26±1.27	95.68±1.98	98.13±0.73	98.31±0.66	96.60±0.79
	+	96.47±0.68	96.61±0.80	96.15±0.79	98.60±0.56	98.61±0.62	98.66±1.33
Effect							
Diet	regular corn	91.66±4.31	90.49±4.97	93.90±3.03	98.45±0.48	98.53±0.58	97.19±0.82
	waxy corn	95.81±2.03	96.44±0.97	95.92±1.46	98.36±0.66	98.46±0.63	97.13±0.77
	-	93.78±3.33	93.76±3.95	94.67±2.65	98.25±0.67	98.45±0.69	96.67±0.72
ABV	+	93.69±4.44	93.17±5.40	95.15±2.51	98.57±0.41	98.54±0.51	98.61±0.39
*P*-value							
Diet		0.008	0.001	0.061	0.705	0.808	0.932
ABV		0.948	0.704	0.641	0.195	0.755	0.030
Deit × ABV		0.329	0.541	0.994	0.533	0.405	0.651

“+”, inclusion of 6.4 g/t virginiamycin in the diet (ABV); “-”, diet without virginiamycin; *P*-value, indicates ANOVA *P*-value; *n* = 6 replicate cages per treatment.

Consistent with the starch digestibility results, broilers fed the waxy corn diet tended to have up-regulated *GLUT2* expression in the jejunum (*P* = 0.051), but no significant effects were observed on the expression of *SGLUT 1* (Table 6). A significant Diet × ABV interaction was observed for Na^+^-*K*^+^-ATPase activity. Specifically, jejunal Na^+^-*K*^+^ATPase activity in chickens fed waxy corn treated with ABV was significantly lower than that in the other treatment groups (*P* < 0.05).

**Table 6:** Relative mRNA expression of jejunal nutrient transporters and Na^+^-*K*^+^-ATPase activity for broiler chickens receiving different dietary corn starches and antibiotics.

Diet	ABV	*SGLT1*	*GLUT2*	Na^+^-*K*^+^-ATPase
Regular corn	-	1.00±0.58	1.03±0.23	1.59±0.30^a^
	+	1.63±0.49	0.70±0.30	1.70±0.21^a^
Waxy corn	-	1.36±0.15	1.55±0.82	1.69±0.34^a^
	+	1.20±0.54	1.15±0.62	1.25±0.04^b^
Effect				
Diet	regular corn	1.32±0.61	0.85±0.31	1.65±0.25
	waxy corn	1.28±0.39	1.35±0.72	1.49±0.33
ABV	-	1.18±0.45	1.31±0.65	1.64±0.31
	+	1.42±0.54	0.93±0.52	1.50±0.28
*P*-value				
Diet		0.842	0.051	0.138
ABV		0.233	0.134	0.122
Deit × ABV		0.082	0.906	0.019

Within a column, means not sharing a common superscript letter are significantly different at *P* < 0.05; “+”, inclusion of 6.4 g/t virginiamycin in the diet (ABV); “-”, diet without virginiamycin; *P*-value, indicates ANOVA *P*-value; *n* = 6 replicate cages per treatment.

The waxy corn diet significantly increased serum triglyceride levels, and antibiotic supplementation also increased serum total triglyceride and glucose (**SGlu**) levels (*P* < 0.05; Table 7). No significant Diet × ABV interaction was observed for the serum parameters (*P* > 0.05).

**Table 7:** Effect of corn starch type and ABV on serum parameters on d 34 broilers (Fasted).

Diet	ABV	SGlu (mmol/L)	TG (mmol/L)	INS (μIU/ml)	GLU (pg/mL)	IGF-1 (ng/mL)
Regular corn	-	8.48±1.10	0.47±0.09	6.86±1.87	104.41±6.67	34.37±4.88
	+	7.94±1.21	0.50±0.08	6.90±2.18	123.06±18.53	31.08±5.44
Waxy corn	-	7.77±1.23	0.50±0.07	7.01±1.42	109.76±22.66	34.51±4.86
	+	7.39±1.12	0.62±0.11	6.68±1.36	142.74±48.51	29.30±8.01
Effect						
Diet	regular corn	8.21±1.14	0.48±0.08	6.88±1.94	113.73±16.47	30.97±8.25
	waxy corn	7.58±1.14	0.56±0.11	6.85±1.34	126.25±40.00	31.91±5.16
ABV	-	8.12±1.17	0.48±0.08	6.94±1.59	107.08±16.17	30.11±6.64
	+	7.67±1.15	0.56±0.11	6.79±1.74	132.90±36.49	34.42±4.64
*P*-value						
Diet		0.210	0.045	0.960	0.295	0.747
ABV		0.349	0.045	0.836	0.038	0.105
Deit × ABV		0.873	0.176	0.796	0.545	0.705

“+”, inclusion of 6.4 g/t virginiamycin in the diet (ABV); “-”, diet without virginiamycin; SGlu, glucose in serum; TG, total triglycerides; INS, insulin; GLU, glucagon; IGF-1, insulin-like growth factor-1; *P*-value, indicates ANOVA *P*-value; *n* = 6 replicate cages per treatment.

### Microbial diversity and composition in the crop

**Fig. 1:**
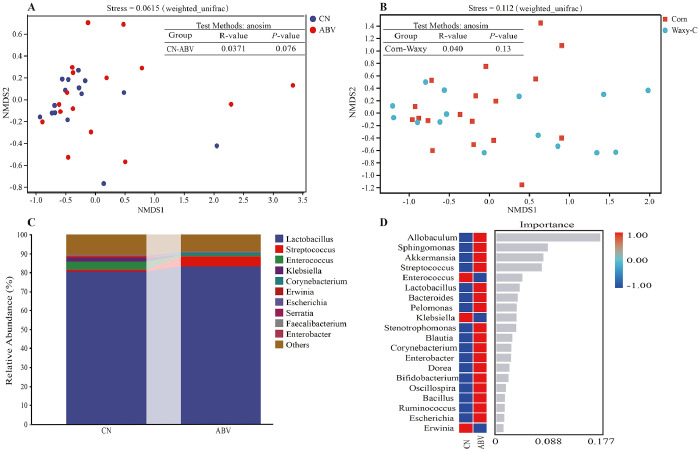
Effects of corn type and ABV on microbial composition in the crop of broiler chickens. A, NMDS analysis between the Con group and ABV group; B, NMDS analysis between the regular corn group and waxy corn group; C, relative abundance of microorganisms at the genus level; D, random forest analysis of microorganisms at the genus level. CN, diet without virginiamycin; AVB, inclusion of 6.4 g/t virginiamycin; Corn: diet based on regular corn; Waxy-C: diet based on waxy corn.

As the microbial data did not meet the assumptions for a two-way ANOVA, the effects of dietary corn type and antibiotic treatment were analyzed separately as the main effects. The observed-species and Chao1 indices reflected community richness, whereas the Shannon and Simpson indices represented community diversity. The dietary corn type had no significant effect on the alpha diversity of the crop microbiota in broilers. However, the inclusion of antibiotics in the diet significantly reduced alpha diversity indices, including Chao1, observed-species, Shannon, and Simpson indices (*P* *<* 0.05; Table 8). Beta-diversity metrics were used to assess the overall differences between treatments and were visualized using NMDS analysis based on the weighted UniFrac dissimilarity matrix. The results indicated that neither dietary corn type nor antibiotic treatment significantly affected the beta diversity of the crop microbiota (*P* = 0.13, *P* = 0.076, respectively; Fig. 1A and B). However, antibiotic treatment showed a *P*-value approaching significance (0.05). Consequently, further analyses were conducted to explore the microbial differences between the antibiotic treatment and control groups. At the genus level, antibiotic treatment increased the relative abundance of *Lactobacillus* and *Streptococcus* in the crop, whereas it reduced the relative abundance of *Enterococcus* (Fig. 1C). Random forest analysis identified *Allobaculum, Sphingomonas, Akkermansia*, and *Streptococcus* as the key differential biomarkers between the antibiotic-treated and control groups (Fig. 1D).

**Table 8:** Effects of corn type and antibiotics on microbial alpha diversity in the crop of broiler chickens.

Alpha diversity^1^	Corn type			ABV		
	Regular corn	Waxy corn	*P*-value	-	+	*P*-value
Chao1	140.91±45.15	179.10±78.36	0.810	191.14±47.9	129.63±61.88	0.040
Observed-species	104.84±30.22	123.42±92.36	0.610	136.76±82.39	92.34±41.22	0.065
Shannon	3.81±0.99	3.43±1.18	0.250	4.26±0.91	3.03±0.91	0.001
Simpson	0.80±0.15	0.76±0.16	0.210	0.85±0.11	0.70±0.16	0.003

“+”, the inclusion of 6.4 g/t virginiamycin in the diet (ABV); “-”, diet without virginiamycin; *n* = 6 replicate cages per treatment.

1 Comparison of group differences using T-Test due to unequal variance precluding two-way ANOVA.

### Microbial diversity and composition in the ileum

**Fig. 2:**
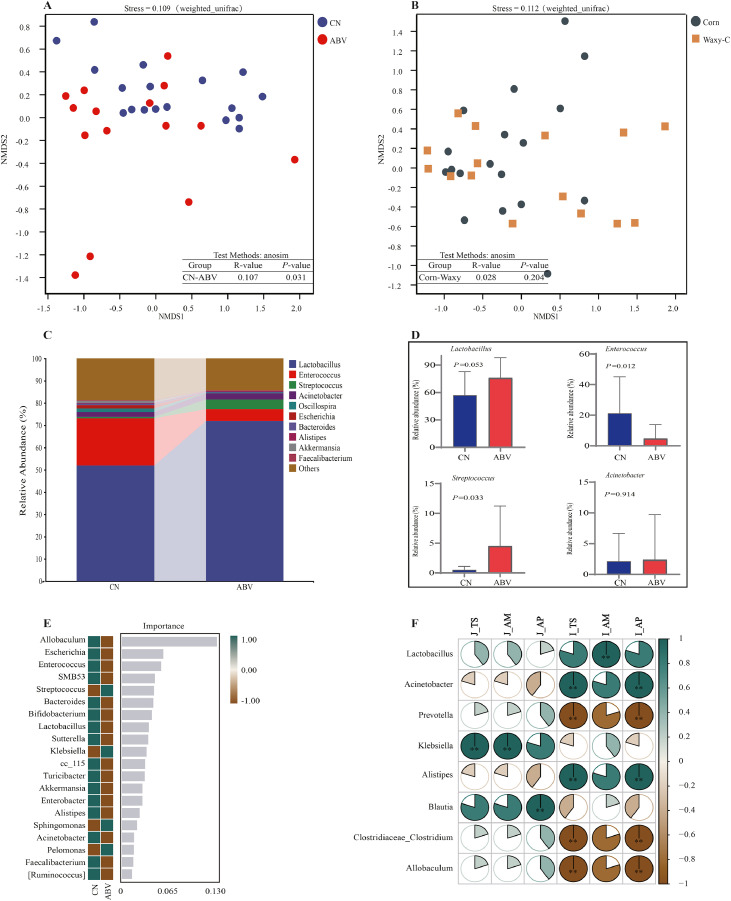
Effects of corn type and antibiotics on microbial composition in the ileum of broiler chickens. A, NMDS analysis between the Con group and ABV group; B, NMDS analysis between the regular corn group and waxy corn group; C, relative abundance of microorganisms at the genus level; D, T-tests for the top 4 relative abundance of microorganisms at the genus levels; E, random forest analysis of microorganisms at the genus level; F, Spearman's correlation analysis between relative abundance of microorganisms and starch digestibility; CN, diet without virginiamycin; AVB, inclusion of 6.4 g/t virginiamycin in the diet; Corn: diet based on regular corn; Waxy-C, diet based on waxy corn; J_TS, total starch digestibility of the terminal jejunum; J_AM, amylopectin digestibility of the terminal jejunum; J_AM, amylose digestibility of the terminal jejunum; I_TS, total starch digestibility in the terminal ileum; I_AM, amylopectin digestibility in the terminal ileum; I_AM, amylose digestibility in the terminal ileum.

The inclusion of antibiotics in the diet significantly reduced Chao1 and observed species indices of the broiler ileal microbiota (*P* < 0.05). However, no significant effects were observed on the Shannon and Simpson indices (Table 9). Antibiotic treatment also significantly influenced the beta diversity of the ileal microbiota (*P* < 0.05, Fig. 2A). In contrast, dietary corn type had no significant effect on either alpha or beta diversity of the ileal microbiota (Fig. 2B). Further analysis revealed that antibiotic treatment increased the relative abundance of *Lactobacillus* and *Streptococcus* in the ileum (*P* = 0.053 and *P* = 0.033) and significantly reduced the relative abundance of *Enterococcus* (*P* < 0.05, Fig. 2C and D). Random forest analysis identified *Allobaculum, Escherichia, Enterococcus*, and *Streptococcus* as the key differential microbiota among the top 20 genera in the antibiotic-treated and control groups (Fig. 2E). Correlation analysis demonstrated that amylose digestibility in the ileum was strongly and positively correlated with the relative abundance of *Lactobacillus.* In contrast, total starch and amylopectin digestibility were highly positively correlated with the relative abundances of *Acinetobacter* and *Alistipes* (*P* < 0.001, Fig. 2F).

**Table 9:** Effects of corn type and antibiotics on microbial alpha diversity in the ileum of broiler chickens.

Alpha diversity^1^	Corn type			ABV		
	Regular corn	Waxy corn	*P*-value	-	+	*P*-value
Chao1	308.50±240.88	236.37±193.15	0.386	373.11±258.14	152.76±91.06	0.030
Observed-species	266.27±202.66	202.68±181.42	0.366	316.43±220.88	137.23±78.95	0.047
Shannon	3.96±0.94	3.27±0.94	0.052	3.80±1.21	3.42±0.61	0.890
Simpson	0.85±0.06	0.76±0.11	0.216	0.80±0.12	0.83±0.07	0.610

“+”, the inclusion of 6.4 g/t virginiamycin in the diet (ABV); “-”, diet without virginiamycin; *n* = 6 replicate cages per treatment.

1 Comparison of group differences using T-Test due to unequal variance precluding two-way ANOVA.

## Discussion

Dietary energy utilization plays a crucial role in animal growth. Starch is an essential primary energy source for this process (Bedford and Apajalahti, 2022). Based on its degree of gelatinization, dietary starch, including amylopectin and amylose, has distinct digestive properties in the gastrointestinal tract (Kljak et al., 2019). Waxy corn, which contains a higher proportion of amylopectin and a lower amount of amylose than regular corn, is more rapidly digestible. Previous studies have suggested that broilers fed diets based on waxy corn exhibit improved feed conversion ratios compared with those fed normal corn (Collins et al., 2003; Luo et al., 2023, 2025). Consistent with these findings, we observed that broilers receiving waxy corn diets had significantly better F/G ratios and higher body weight gain. The differences in growth performance associated with the corn type may be attributed to the digestibility of corn starch. Indeed, higher starch digestibility is commonly linked to improved feed conversion efficiency, as reported in several studies (Del et al., 2009; Wang et al., 2023).

Antibiotic growth promoters (**AGP**) are a class of antimicrobial feed additives that are administered to animals at sub-therapeutic doses to enhance growth (Broom, 2017). Despite being banned in many countries, AGP continues to significantly affect animal growth performance. Understanding the complex mechanisms underlying the growth-promoting effects of AGP is essential for the development of effective alternatives. Previous studies have suggested that the beneficial effects of antibiotics on animal performance may stem from the modulation of the gut microbiota, enhancement of the mucosal barrier, regulation of the immune system, inhibition of pathogen colonization, and influence on energy metabolism (Lange et al., 2016; Broom, 2018; Zhu et al., 2021). The antibiotic virginiamycin (ABV) was widely used in animal production. Research showed that ABV improves FCR and increases cecal propionate levels in broilers (Pourabedin et al., 2015). In the present study, ABV supplementation significantly improved the F/G ratio of broiler chickens. ABV improves nutrient utilization, including energy, crude protein, and amino acids, thereby enhancing the growth performance of livestock (Agudelo et al., 2007; Stewart et al., 2010).

To further investigate the potential mechanisms underlying the effects of corn type and antibiotic treatment on broiler growth performance, we examined their effects on starch digestibility. In the crop, the total starch content was unaffected by either corn type or antibiotic treatment, suggesting limited starch digestion and absorption in this region. Notably, the proportion of amylopectin in the crops of chickens fed with waxy corn was lower than that in the feed. Despite this reduction, the remaining amylopectin content was still significantly higher than that in the regular corn group, indicating a more complete degradation of amylopectin in the crops of birds that received waxy corn. Furthermore, a significant Diet × ABV interaction was observed for both amylopectin content and amylopectin/amylose ratio in the crop. Specifically, the amylopectin content in the crops of the waxy corn-ABV group was significantly lower than that in the waxy corn-without ABV group, suggesting potential antibiotic-mediated modulation of amylopectin degradation. Previous studies have proposed that the crop primarily serves to store and moisten food through microbial fermentation (Saxena et al., 2016; Wu et al., 2020). Changes in amylopectin levels during this process may be influenced by microbial activity of the host. It is also worth noting that this outcome may be influenced by digesta retention time in the crop, which could introduce confounding variables in analytical methodologies for amylopectin/amylose quantification. While these findings highlight the complex interplay between microbial ecology and nutrient dynamics, further research is warranted to elucidate the underlying mechanisms involved. In contrast, our data indicate that starch digestibility approaches approximately 90 % in the distal jejunum. Broilers fed waxy corn diets exhibited significantly higher total starch and amylopectin digestibility than those fed regular corn diets, whereas the antibiotic treatment had no significant effect on starch digestibility. Although microbial starch processing occurs in crops, it does not extend into the jejunum. The majority of starch digestion and glucose absorption in broilers occurs within the duodenum and jejunum during a brief retention time of approximately 1 h (Rougiere and Carre, 2010), highlighting the critical role of the jejunum in starch digestion. Starch digestion at this stage is primarily facilitated by host-secreted enzymes, making the dietary starch composition a key factor influencing starch digestibility in the jejunum. Studies have demonstrated that starch granule size varies among wheat, sorghum, and corn, with corresponding differences in apparent starch digestibility (0.85 vs*.* 0.84 vs*.* 0.90) in the terminal jejunum of broilers at 35 d of age (Liu et al., 2014). Even within the same grain, varying the proportions of amylopectin and amylose significantly affected starch digestibility. Compared with amylose, amylopectin, which has a higher degree of branching, larger molecular surface area, and weaker intermolecular hydrogen bonds (Yang et al., 2023), is more easily digestible. This was evident in the present study, in which broilers fed waxy corn exhibited significantly higher starch digestibility in the jejunum. Other studies have also reported higher starch digestibility in waxy corn owing to its higher amylopectin content (Akay and Jackson, 2001; Ma et al., 2019). Notably, the broilers fed waxy corn diets in our study also showed a marked increase in *GLUT2* expression in the jejunum, suggesting that enhanced starch digestibility in waxy corn may be linked to improved glucose transport. Glucose derived from starch is primarily transported into enterocyte by glucose transporters, and elevated glucose concentrations in the intestinal lumen induce the upregulation of *GLUT2* expression on the brush border membrane, thereby enhancing glucose absorption (Schmitt et al., 2017; Primec et al., 2021). As most starch is already digested and absorbed in the jejunum, no significant effect of corn type on starch digestibility was observed in the distal ileum. This indicates that the effect of corn type on broiler growth performance is mediated primarily by enhanced starch digestion in the jejunum. Interestingly, the addition of antibiotics significantly increased amylose digestibility in the distal ileum. Amylose has a longer retention time in the digestive tract due to its lower branching and more stable crystalline structure, with some indigestible portions reaching the large intestine for microbial fermentation. The ileum is a rich microbial community that plays crucial roles in maintaining gut health, modulating immunity, and facilitating nutrient digestion. The increased starch digestibility in the terminal ileum induced by antibiotics may indicate a role for the ileal microbiota, particularly amylose, in starch digestion. However, the relationship between dietary nutrients and the gut microbiota remains complex. Whether antibiotics directly alter microbial populations and influence starch digestibility or whether nutrient availability drives microbial shifts requires further investigation. In addition, changes in starch digestibility were accompanied by notable alterations in serum parameters. Both waxy corn and antibiotic treatments elevated the serum triglyceride levels, suggesting enhanced lipid metabolism in broilers. Antibiotic treatment also significantly increases serum glucagon levels, which are critical for regulating glucose, lipid, and amino acid metabolism, potentially explaining the growth-promoting effects of antibiotics in poultry.

The poultry gut microbiota can reach densities of up to 10¹¹ organisms per gram (Wei et al., 2013), forming a complex microbial community that is essential for regulating gut health (Yang et al., 2024) and enhancing nutrient utilization (Koh et al., 2016). In the crop, the dominant microbial species included *Lactobacillus, Enterococcus*, and *Streptococcus*, with *Lactobacillus* being the most prevalent, ranging from 1 × 10⁸ to 1 × 10⁹ cfu/g. *Lactobacillus* primarily breaks down starch, ferments lactic acid, inhibits pathogenic bacteria, and maintains microbial balance in the crop (Apajalahti and Vienola, 2016). Our findings also indicated that *Lactobacillus* had the highest relative abundance in the broiler crop. Antibiotic treatment suppressed the growth of certain microbes, reduced the alpha diversity of the crop microbiota, and increased the relative abundance of *Lactobacillus* and *Streptococcus*, although these differences were not statistically significant. Interestingly, antibiotics exerted a similar effect in the ileum, where they reduced the Shannon and Simpson indices and significantly affected beta diversity. At the genus level, antibiotics significantly increased the relative abundance of *Streptococcus* and *Lactobacillus*, and decreased the relative abundance of *Enterococcus*. Research has shown that ABV supplementation increases the body weight of 42-day-old birds, coinciding with an increase in *Lactobacillus* abundance in the ileum (Ramiah et al., 2014). However, few studies have explored the relationships among ABV, gut microbial metabolites, and poultry growth. Feng et al. identified *Lactobacillus* as the dominant microbe in the ileum, followed by *Streptococcus* and *Helicobacter*, with *Enterococcus* showing a higher relative abundance in early life (1 to 4 days old) but declining after day 28 (Feng et al., 2023). By contrast, the control group exhibited a higher relative abundance of *Enterococcus* in the ileum. *Enterococcus* is often considered a potential pathogen in poultry and is capable of causing diseases such as endocarditis and spondylitis under certain conditions (Jung et al., 2018). Conversely, *Lactobacillus* maintains gut health and promotes animal growth (Spivey et al., 2014). This study also found a significant positive correlation between *Lactobacillus* abundance and ileal starch digestibility. Notably, the type of corn used in the diet did not significantly affect microbiota diversity in either the crop or the ileum, likely because different corn types did not cause notable changes in ileal starch digestibility, meaning that the nutrients available to microbes remained largely consistent. In summary, these microbial findings suggest that antibiotics enhance ileal starch digestibility by increasing the abundance of beneficial bacteria associated with starch digestion and reducing harmful bacteria, ultimately improving growth performance.

## Conclusion

The type of corn and inclusion of virginiamycin in the diet influenced starch digestibility, which in turn affected growth performance. Increased amylopectin content in waxy corn enhances starch digestibility in the jejunum, contributing to growth performance. In contrast, antibiotics improved the amylose digestibility in the distal small intestine by boosting the relative abundance of starch-digesting microbes (*Lactobacillus*). The specific metabolic mechanisms through which these microbes promote starch digestion, as well as the underlying interactions between corn type and virginiamycin in broilers require further investigation. This study underscores the critical role of corn type and antibiotic-mediated changes in the gut microbiota in enhancing intestinal starch digestibility in broiler chickens, providing valuable insights into exploring new alternatives to AGP.

## Funding

This research was supported by the National Key R&D Program of China (2021YFD1300404); the Fund Program for Scientific Activities of Selected Returned Overseas Professionals in the Shanxi Province (20220016); and Earmarked Fund for Modern Agro-industry Technology Research System of Shanxi province (2024CYJSTX15).

## Informed consent statement

Informed consent was obtained from all subjects involved in the study.

## CRediT authorship contribution statement

**Yanwei Xu:** Writing – original draft. **Yong Xiao:** Formal analysis. **Chunxiao Ai:** Data curation. **Yihong Huang:** Methodology. **Huajin Zhou:** Investigation. **Jianhui Li:** Writing – review & editing. **Jianmin Yuan:** Project administration.

## Declaration of competing interest

The authors declare that they have no known competing financial interests or personal relationships that could have appeared to influence the work reported in this paper.

## Appendix Supplementary materials

Image, application 1

Image, image 2
