# Supplementary figures and images for: Antibiotic growth promoters and waxy corn enhance broiler growth performance through starch digestibility and microbiota modulation in the crop and ileum
Source: Poult Sci. 2025 May 10;104(8):105288. doi: 10.1016/j.psj.2025.105288 (PMC12148569; doi:10.1016/j.psj.2025.105288)

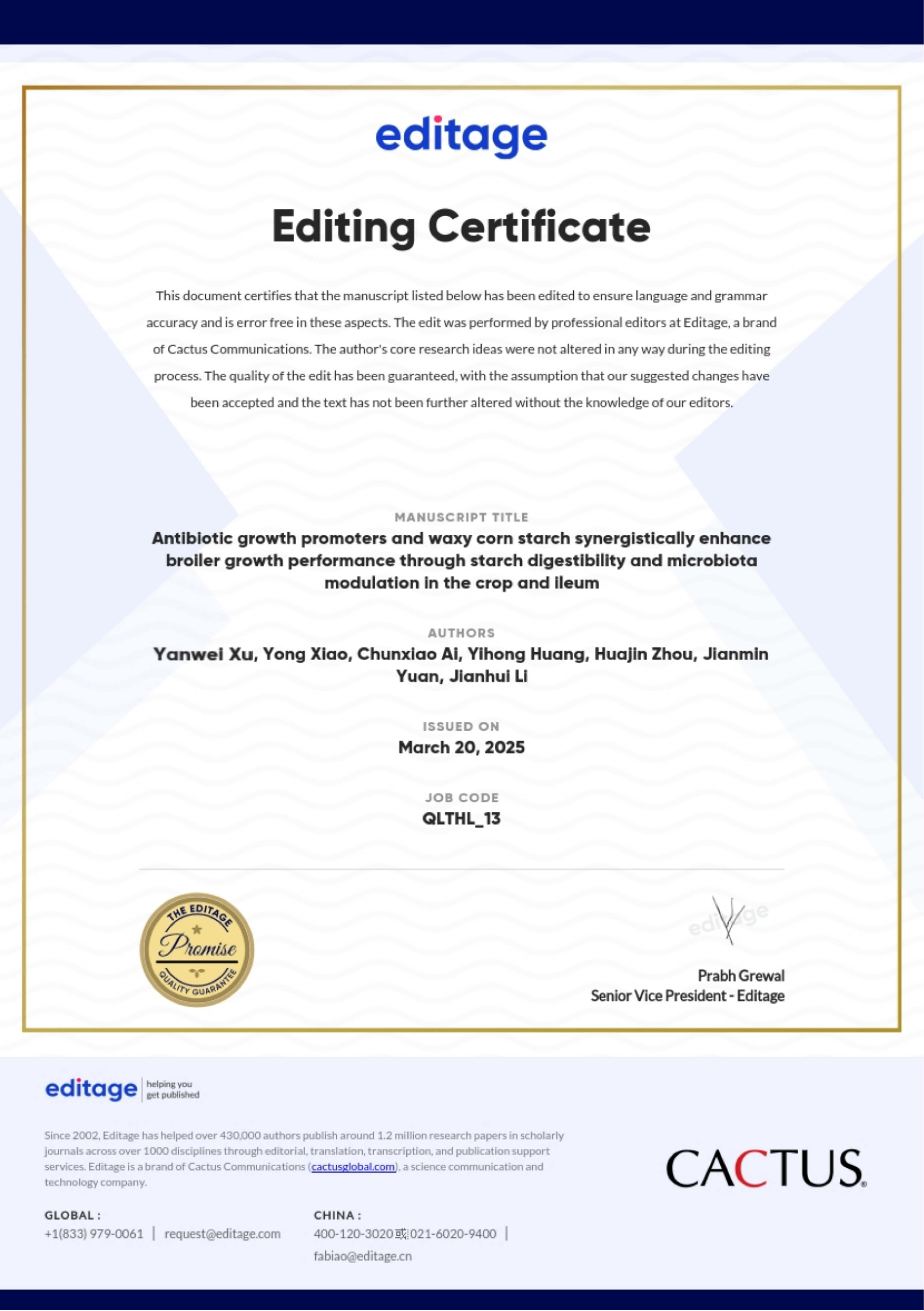

Supplement: Supplementary file 2 [file mmc2.jpg]
