# Supplementary material for: Antibiotic growth promoters and waxy corn enhance broiler growth performance through starch digestibility and microbiota modulation in the crop and ileum
Source: Poult Sci. 2025 May 10;104(8):105288. doi: 10.1016/j.psj.2025.105288 (PMC12148569; doi:10.1016/j.psj.2025.105288)
Supplement: Supplementary file 1 [file mmc1.pdf]

Changes to authorship

Article Entitled: Antibiotic growth promoters and waxy corn starch synergistically enhance broiler growth performance through starch digestibility and microbiota modulation in the crop and ileum

Author: Yanwei Xu[a,b], Yong Xiao[a], Chunxiao Ai[a], Yihong Huang[a], Huajin Zhou[a], Jianhui Li[b], Jianmin Yuan[a]*

[a]State Key Laboratory of Animal Nutrition and Feeding, College of Animal Science and Technology, China Agricultural University, Beijing 100193, China

[b]College of Animal Science, Shanxi Agricultural University, Taigu 030801, China

Dear Editor:

I am writing on behalf of all authors of the manuscript titled " Antibiotic growth promoters and waxy corn starch synergistically enhance broiler growth performance through starch digestibility and microbiota modulation in the crop and ileum" (Submission ID: **PSJ-D-25-00590**) to formally request an adjustment to the authorship list and corresponding author designation. After thorough discussion and mutual agreement among all authors, it has been decided that Dr. **Jianmin Yuan** should be designated as the sole corresponding author in place of Dr. **Jianhui Li.** Both Dr. **Jianmin Yuan** and Dr. **Jianhui Li** have made equally significant contributions to the theoretical and technical guidance, as well as providing financial support for the work. However, as the journal's policy does not permit co-corresponding authors, all authors have agreed that Dr. Jianmin Yuan will serve as the sole corresponding author.

Additionally, I would like to confirm that all authors have provided their written consent signed letters, agreeing to this change in authorship and the rearrangement of the author list. Below are the signatures of all authors.

Signed: Yanwei Xu, Yong Xiao, Chunxiao Ai, Yihong Huang, Huajin Zhou, Jianhui Li, Jianmin Yuan

We sincerely apologise for any inconvenience this amendment may cause and deeply appreciate your understanding in facilitating this update. Please do not hesitate to contact me or Dr. Jianmin Yuan should further documentation or clarification be required. Thank you for your time and consideration.

Jianhui Li Jianhui Li

On behalf of all authors
